# Who Volunteers? Results from a Regular Cognitive Monitoring Study

**DOI:** 10.1093/geroni/igab046.2595

**Published:** 2021-12-17

**Authors:** Britney Veal, Nasreen Sadeq, Taylor Atkinson, Ross Andel

**Affiliations:** University of South Florida, Tampa, Florida, United States

## Abstract

Previous research indicates volunteering promotes well-being of individuals and communities. Volunteering in later-life may buffer some of the negative health effects experienced during retirement, facilitating opportunities for older adults to engage in meaningful activities and stay active. The current study examined characteristics of older adults who volunteered outside of participation in a regular cognitive monitoring study. All 124 members (M= 76.87, SD= 7.47; 80 volunteers, 44 non-volunteers) of a regular cognitive monitoring study, requiring completion of a 15-minute cognitive online test once a month, with complete data on personal characteristics, volunteer activities, as well as study adherence and dropout rates were included. ANCOVA and logistic regression analyses adjusted for sociodemographic characteristics were used to assess differences between volunteers and non-volunteers. Results indicated that volunteers were less educated (p<.05), and slightly more likely to be younger and women compared to non-volunteers. There were no differences in cognitive performance (ps>.05). Volunteers had lower scores for neuroticism (p=.02) and were marginally higher agreeable and extraverted (ps<.09). Volunteers needed more reminders to complete the monthly test (ps<.01) but had lower dropout rates (p=.001). The most frequent type of volunteer activity reported was religious. Volunteers were motivated mainly by altruism, although most reported multiple reasons such as building social relationships and feeling important. Findings provide information about characteristics that can help identify older adults who are likely to volunteer. Results regarding study adherence may have implications for promoting recruitment and retention among older adult volunteers.

